# Using Structural Equations to Model the Relationships between Procedural Justice, Risky Lifestyles, and Violent Inmate Misconduct

**DOI:** 10.3390/ijerph17217927

**Published:** 2020-10-29

**Authors:** Jaeyong Choi, Glen A. Ishoy, Julak Lee

**Affiliations:** 1 Department of Security Studies and Criminal Justice, Angelo State University, San Angelo, TX 76901, USA; jaeyong.choi@angelo.edu; 2 Department of Criminology and Criminal Justice, Indiana University of Pennsylvania, Indiana, PA 15705, USA; glen.ishoy@iup.edu; 3 Department of Industrial Security, Chung-Ang University, Seoul 06974, Korea

**Keywords:** prisoners, violent inmate misconduct, procedural justice, risky lifestyles

## Abstract

Prior research has consistently shown that perceptions of procedural justice promote individuals’ compliance with the law. Several studies have also identified mechanisms that explain the association between perceptions of procedural justice and compliance (e.g., social identity). However, the potential role of risky behaviors as a mediator of the association between procedural justice and compliance remains unexplored. This study examined whether risky behaviors can mediate the relationship between procedural justice and violent inmate misconduct. Data for this study were derived from a sample of 986 incarcerated felons in South Korea. The present study employed structural equation modeling to test how risky lifestyles mediate the association between procedural justice and violent misconduct. The results showed that procedural justice reduced violent inmate misconduct. Additionally, the mediation hypothesis received partial support: the direct effect of procedural justice on violent misconduct was partially mediated by involvement in risky activities. Taken together, the results highlight the importance of the interrelationship between procedural justice, risky lifestyles, and violent misconduct in a prison setting.

## 1. Introduction

It is well documented that the impact of procedural justice can be more effective than a deterrence-based model of regulation in terms of encouraging citizens to obey the law and its legal agents [1,2,3,4,5]. When citizens perceive that they are being treated fairly, they are more likely to comply with the law [6,7]. Research evidence has consistently supported the efficacy of procedural justice in gaining compliance from offender populations, e.g., [8,9,10].

According to procedural justice theory, when people experience procedurally fair treatment at the hands of legal actors, especially the police, the experience of fairness reinforces their sense of belonging to the social groups that legal agents represent, strengthening the bond of societal norms and values [11,12]. Several empirical studies have shown that procedural justice is related to how individuals view their social identity, which ultimately influences their perceived duty to obey the law [13,14,15]. Some researchers emphasized the role of negative emotions (e.g., anger) in linking procedural injustice to noncompliance, based on the equity theory [16]. This perspective posits that people can be motivated to become noncompliant with authorities’ rules when they perceive that they are being treated unfairly because they want to engage in retaliatory behavior to restore equity [17].

Although there are some differences, much of the research on procedural justice has focused on the normative value of procedural justice [18,19,20]. This approach emphasizes a relational notion of identity, suggesting that people who experience positive signs and symbols of their worth and standing are more likely to internalize group values [2,21]. Internalizing group values increases the normative constraints on behavior [14]. Although this perspective has received much attention and has found strong empirical support [22], the literature on the procedural justice theory has not widely considered the potential of opportunity perspectives to explain the observed relationships between procedural justice and violent misconduct in the incarceration setting [23,24,25]. Specifically, procedurally fair treatment by criminal justice practitioners may effectively reduce serious forms of crime by reducing participation in risky behaviors. If fair treatment promotes self-worth and value within the group [26], this may discourage individuals from engaging in risky behaviors that can expose them to “high-risk times, places and people” [27]. Thus, the current study aims to explore the links between procedural justice, risky behaviors, and violent inmate misconduct using data from a sample of South Korean inmates.

## 2. Literature Review

A large body of survey-based work has shown that perceptions of procedural justice are directly and indirectly associated with greater compliance with the law and rules, e.g., [28,29,30]. Moreover, risky lifestyles within the incarceration setting are often illegal activities (e.g., participation in gambling) [31,32]. Accordingly, the argument we advance in this article is two-prong. First, when incarcerated felons perceive that they have received unfair treatment, they are more likely to not comply with the rules—resulting in more frequent participation in illegal activities in the prison setting. Second, the increased risky lifestyles can lead to more violent inmate misconduct due to the interpersonal conflicts that arise from risky lifestyles. Our propositions are plausible considering a fair amount of research on the link between procedural justice and rule-breaking behaviors. Therefore, we review separate strands of literature pertaining to procedural justice, risky lifestyles, and inmate misconduct.

### 2.1. Procedural Justice as an Antecedent of Risky Lifestyles

The opportunity perspective stresses the opportunistic nature of crime. According to Cornish and Clarke [33], offenders make decisions just like everyone else. What drives their decision to commit a crime is based on a specific setting that can maximize pleasure and minimize pain [34]. The favorable settings for motivated offenders involve a suitable target and an opportune time/place context [23]. Consistent with this perspective, researchers have considered the importance of situations that are conducive to crime in addition to other prominent criminological concepts, such as peer socialization and criminality [35,36,37,38]. This line of inquiry has documented that situational opportunity is important in understanding crime and delinquency [25,39].

The two most prominent theories regarding the opportunity perspective are the lifestyle theory and the routine activities theory [23,27]. Despite some notable differences [40], researchers have considered these theories as components of one broader theoretical perspective because both theories emphasize behavioral patterns of likely offenders and potential victims instead of criminality, which is a focus of traditional criminological theories. The routine activities theory posits that a crime event will occur when three elements converge: (1) the presence of a suitable target, (2) the presence of a motivated offender, and (3) the absence of guardianship [25]. Thus, as likely offenders encounter potential victims while there is no appropriate guardianship, the risk of a crime event increases. The lifestyle theory also underlines that some lifestyles are more likely to increase the risk of being a crime victim [27].

The lifestyle and routine activities theories successfully explained different types of crime events, whether online and offline [41,42,43]. Although early studies drawing on these theories tended to focus on crime rates or victimization as outcomes, scholars began to extend the notion of opportunity to account for individual offending [44]. A considerable body of research has documented that measures of deviant behaviors and offending are associated with one’s risky lifestyle choices, particularly one’s involvement in unstructured activities without supervision [35,36,45,46]. Based on this ample empirical evidence, we suggest that inmates who are frequently involved in unstructured activities in the incarceration setting are more likely to engage in violent misconduct.

Scholars have found that certain individuals are more likely to participate in risky lifestyles and put themselves in a situation conducive to the criminal event, e.g., [47,48,49,50]. Studies have repeatedly examined the importance of individual differences with regard to situational opportunity within the prison context, but most of these studies mainly focused on individual traits, such as low self-control, that lead some people to adopt risky lifestyles [51,52,53]. For example, one study linked self-control to inmate deviance while considering the religious involvement of prisoners [32]. Kerley, Copes, Tewksbury and Dabney [32] found that the relationships between religious behaviors and prison deviance were significant and that some of the relationships became insignificant once self-control was included in the model. Then, they performed a regression of self-control on three religious behaviors (i.e., watching religious television, attending religious events, and praying privately). They found that self-control was a significant predictor of all three religious behaviors.

However, research has yet to assess the extent to which perceived procedural justice affects inmates’ risky activities, which consequently impacts the likelihood of engaging in violent misconduct. In the next section, we leverage information from previous empirical studies to create a set of hypotheses linking procedural justice, risky activities, and violent misconduct.

### 2.2. Procedural Justice and Inmate Violent Misconduct

The argument we test in the current study is that inmates with high perceptions of procedural justice are more likely to comply with the decisions of authorities and institutional rules and that this may discourage them from participating in risky behaviors, which reduces their involvement in serious crimes. Procedural justice literature reveals that people who feel that they are being treated fairly tend to obey the law [1,22]. Within the correctional context, we believe that there is a reason why inmates would be more compliant with institutional regulations when inmates feel they are being treated fairly. Specifically, procedural fairness can promote group membership [14]. Because correctional officers may represent society’s prototypical moral values, the behavior of the correctional officer provides information to the inmate about their value and social standing [54]. When correctional officers treat inmates with dignity and respect, the inmates feel respected and valued by the group, which enhances self-regulating beliefs [55,56,57].

Although the overall conclusion that procedural justice is linked to compliance with the law has been consistently found in different settings and sociopolitical contexts [58,59,60,61], studies examining the importance of procedural justice within prison settings remain, however, limited, see (although see, [62,63,64,65,66,67]). Three recent studies concerning the relationship between procedural justice and inmate misconduct are particularly important. In the first, Reisig and Meško [64] used data from an adult prison in Slovenia to examine the relationship between perceived procedural justice and prisoners’ misconduct. Most notably, their analysis showed that inmates who viewed correctional officers as procedurally impartial self-reported less misconduct, and fewer rule infractions were recorded among inmates with higher perceived procedural justice.

In a second study, Beijersbergen, Dirkzwager, Eichelsheim, Van der Laan and Nieuwbeerta [63]—drawing on data from a longitudinal, nationwide study in the Netherlands—used cross-lagged structural equation models to examine the longitudinal associations between procedural justice and inmate misconduct. They found that perceptions of procedural justice were predictive of fewer incidents of inmate misconduct and disciplinary reports. Additionally, they found that anger influenced the association between procedural justice and prisoners’ misconduct. Importantly, these authors did not investigate how procedural justice can influence risky activities in prison settings and how these risky activities are related to more serious forms of misconduct. Last, and the study we build on in the current analysis, Choi [65] used data from a sample of South Korean inmates and investigated whether procedural injustice serves as a strain to promote the risk of violent and nonviolent misconduct. His regression models revealed that perceived procedural injustice is associated with both violent and nonviolent misconduct.

These studies notwithstanding, assessing the differences in the perceptions of procedural justice regarding involvement in risky routines and inmate misconduct is virtually non-existent and deserves empirical scrutiny, especially given the significance of risky behaviors in relation to the occurrence of crime [25,68,69]. The limited research suggests that inmates with low levels of procedural justice are more likely to be involved in risky behaviors, which can promote the likelihood of violent misconduct by presenting settings that are conducive to crime. In sum, we expect that perceptions of procedural justice are negatively associated with risky lifestyles and that risky lifestyles are positively related to violent misconduct. Given that procedural justice can directly affect violent misconduct, it remains unclear whether risky lifestyles fully mediate the relationship between procedural justice and violent misconduct.

### 2.3. The Current Study

Although the field of criminology has produced extensive literature regarding the impact of procedural justice on individuals’ compliance with the law, scholars have not expanded the focus of how procedural justice links to compliance to consider the important insights from the situational opportunity perspectives [25,27]. If inmates with low levels of perceived procedural justice are more likely to be involved in risky behaviors, and if these activities create settings in which inmates are likely to partake in violent offending, the effects of procedural justice on violent offending could be seen as direct and indirect. We argue that there are three theoretical reasons to infer that risky behaviors can increase the likelihood of violent misconduct. First, risky behaviors can entice inmates to attack others. If inmates engage in gambling or illegal substance use in prison, it attracts offenders because these activities involve the proceeds of crime. Potential offenders may want to loot cash or substance for their interests [25]. Second, involvement in risky behaviors can set up another crime. Potential offenders in prison may approach the settings where other inmates participate in risky activities thinking only about cash. However, if they see illegal substances, they may also decide to steal them because they are valuable in the correctional context. Third, risky activities can escalate into violent offending. When the potential offender tries to steal cash or prohibited items from their fellow inmates, the disputes can escalate into violent crime. Therefore, this article seeks to examine the mediating role of risky activities in the association between procedural justice and violent misconduct. We hypothesize that perceived procedural justice is inversely associated with participation in risky activities and that risky routines are positively correlated with violent misconduct.

## 3. Methods

### 3.1. Sample

The data used for the current study came from a research project that was the result of a collaboration between the Korea Correctional Service (KCS) in the Ministry of Justice and Kyonngi University in South Korea. A sample of 986 incarcerated male offenders was surveyed in 2009. Stratified sampling was used by considering several characteristics of prisons in South Korea: the size of prison (housed inmate population), the types of prison (first-time offenders vs. repeated offenders), and the geographic distribution of prison (four regions in South Korea). Subjects were solicited from 20 Korean correctional facilities. Specifically, the research team first selected 13 prisons that housed more than 1000 inmates and seven prisons that held less than 1000 inmates, among 31 male prisons in South Korea. They then randomly selected inmates who had served for more than one year from each institution, considering its inmate capacity. From the prisons that housed more than 1000 inmates, 60 adult male inmates were selected, whereas 40 adult male inmates were selected from the prisons that held less than 1000 inmates. The research team administered a group survey within each facility from 27 July to 13 August in 2009. The total sample size was 986 (Because not all inmates who were randomly selected from the stratified sampling agreed to participate in a self-administered survey, the final sample is smaller than the size of the sample initially chosen). The survey was anonymous, and participation in the survey was voluntary. Details regarding the rationale and overall design of the study can be found in Yoon [70]. The data we drew on in this article have been used in several empirical studies [31,65,71,72,73,74], but have not been used to test how risky lifestyles mediate the association between procedural justice and violent misconduct. Table 1 shows the descriptive information of participants.

### 3.2. Measures

#### 3.2.1. Dependent Variable

The dependent variable in this study is the subject’s self-reported violent misconduct. Three types of misconduct were considered. Subjects were asked how often they have engaged in the following rule violations during the past year: “fighting with fellow inmates,” “assaulting fellow inmates,” and “assaulting correctional officers.” Item responses ranged from 0 (never) to 4 (10 or more times) (We did not recode these responses. The responses to these items were originally categorical). These three indicators were treated as a latent variable, and the one-factor measurement model indicated a good fit to the data. All item loadings on the latent factor were found to be statistically significant (item loadings from 0.43 to 0.90). Table 1 displays the descriptive statistics for the three items.

#### 3.2.2. Independent Variables

The procedural justice scale includes two questions about the respondent’s endorsement of the following statements: “Prison officers are trying to help us” and “Prison officers treat inmates in a respectful and fair manner.” These items capture two elements of the concept of procedural justice discussed in prior research: trustworthiness and respect [20,55] (Our study uses the measure of procedural justice that captures perceptions, not necessarily objective reality. Even though we used the items asking inmates about their experiences with correctional officers, these are not the independent assessment of actual treatment received. Ultimately, inmate perceptions of interactions with correctional officers may not be perfectly correlated with the amount of procedural justice that they actually received, see also [75]). Trust in legal actors and their motivations to do their job have been identified as essential components in the procedural justice theory [1,76]. Simultaneously, the experience of procedurally fair treatment with respect at the hands of a correctional officer can foster positive perceptions of the prison because it communicates information about officers’ democratic values and social standing to inmates, see also [77]. Both items were answered on Likert-type scales from 1 (strongly disagree) to 4 (strongly agree).

#### 3.2.3. Mediator: Risky Lifestyles

Risky lifestyles have been measured in various ways [27,78,79]. In the current study, following recommendations by Pratt and Turanovic [40], we used the measure of risky activities that can capture “*high risk*, people and places” (p. 339). Four questions served as indicators of one’s involvement in risky activities. Respondents were asked to indicate how often they have engaged in the following incidents within the previous year: “Possession of prohibited item,” “having broken away from the designated area,” “having gambled,” and “having participated in transaction” (see also [31]). Each item was rated using a four-point Likert-type scale, from 0 (never) to 4 (10 or more times). When all four items were summed to create an overall metric measuring risky lifestyles, almost half of the answers (49%) on the scale were zero, making it non-normally distributed with high skewness (4.8) and kurtosis (29.55). To better fit the data in the model and deal with non-normality, each item was transformed and dummy coded (coded as 0 = never and 1 = yes). All item loadings on the latent construct were statistically significant (item loadings from 0.57 to 0.63).

#### 3.2.4. Other Covariates

The last set of covariates dealt with the participant’s demographic characteristics (e.g., age, education, and marital status), and the length of time served. To measure instrumental provision supplied by the prison [80], the analysis included whether respondents had participated in the four types of institutional programs (i.e., academic education, vocational training, psychological training, and working in prison). These types of programs are expected to have protective effects because they cannot only help inmates adjust to prison life [81,82] but also provide supervised interactional settings [35,36,69]. Subjects were asked if they had participated in four types of institutional programs during the past year, including academic education, vocational training, psychological training, and working in prison. The response options for each item were 0 (never) and 1 (yes).

### 3.3. Analytic Strategy

Structural equation modeling (SEM) is used for this study. SEM allows for a combination of confirmatory factor analysis (CFA) and path analysis in one model. SEM has some strengths over summing up scales and separate path analysis because SEM can more effectively take into account measurement errors [83]. Creating additive scales and performing an ordinary least squared (OLS) regression assumes that loadings for the factor are equal, which can bias the estimates. Weighted least squares with robust standard errors and a mean- and variance-adjusted chi-square (WLSMV) is employed as the estimator method because of its applicability for dichotomous or categorical variables involved in latent constructs [84]. Likert-type scales with five or fewer items in their responses are typically considered as categorical data [85]. To explore the patterns of missing data, Little’s MCAR test was conducted using R with the “BaylorEdPsych” package (0.5), but the result indicated that missing cases are not completely random. After conducting SEM with both pairwise and listwise methods, the listwise method was used because both models were substantially identical. All analyses were performed using R (ver. 3.5.3), and SEM was conducted with the “lavaan” package (0.63–3).

To evaluate the measurement and structural models, several goodness of fit indices such as chi-square (*χ*^2^) statistics, the Jöreskog and Sörbom goodness-of-fit index (GFI), the Bentler comparative fit index (CFI), and root mean square error of approximation (RMSEA) are used. Since the *χ*^2^ test is sensitive to sample size and larger samples can make *χ*^2^ significant [86], other goodness of fit indices are employed to assess the model [83,87]. If the GFI is higher than 0.95, the model fit is considered acceptable [88]. The goodness of fit is considered appropriate if the CFI is higher than 0.97, whereas the CFI higher than 0.95 is deemed acceptable [89]. If the RMSEA is lower than 0.08, the model fit is considered good, and if the RMSEA is lower than 0.05, the model fit is considered ideal [90]. Overall, the current study estimates the direct and indirect effects of procedural justice on violent misconduct with risky lifestyles as a mediating variable.

## 4. Results

First, we performed CFA to examine the quality of the measurement model. The measurement model passed all the necessary thresholds for goodness of fit: *χ*^2^ = 28.832, df = 24, *p* = 0.226; GFI = 0.997; CFI = 0.987; and RMSEA = 0.015, 95% CI = [0.000, 0.032]. Therefore, the results indicated that the model fit the data well. Table 2 shows that the factor loadings were all statistically significant. In other words, all observed indicators were statistically significant to explain their underlying latent constructs. Additionally, procedural justice was moderately correlated with risky lifestyles and violent misconduct: correlations between procedural justice and violent misconduct (*r =* −0.232, *p* < 0.001) and between procedural justice and risky lifestyles (*r* = −0.218, *p* < 0.001). Notably, the correlation between risky lifestyles and violent misconduct was strong (*r* = 0.578, *p* < 0.001), supporting the argument that one’s lifestyle choices can affect the opportunity to offend by creating convergent spaces where potential offenders and victims meet [25,44].

**Figure 1:**
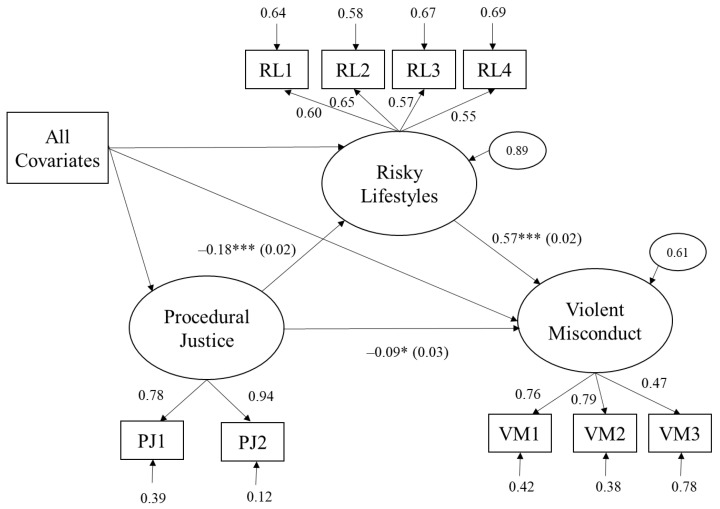
The structural model with standard coefficients. All the covariates were included but shown with a single rectangle. Standard errors for regression coefficients were shown in parentheses. χ^2^ = 138.445, df = 72, *p* < 0.001; GFI = 0.979; CFI = 0.984; and RMSEA = 0.029, 95% CI = [0.015, 0.043]. PJ1 = trustworthiness; PJ2 = respect; RL1 = possessed prohibited items; RL2 = broke away from the designated area; RL3 = gambled; RL4 = transaction; VM1 = fighting with fellow inmates; VM2 = assaulting fellow inmates; and VM3 = assaulting correctional officers. GFI = goodness-of-fit index; CFI = comparative fit index; TLI = Tucker–Lewis index; RMSEA = root mean square error of approximation; and CI = confidence interval. * p < 0.05, *** *p* < 0.001.

After establishing the validity of the measurement model, the full SEM model was constructed, adding all relevant covariates to the model. Regressions were included in the model based on the theoretical framework. In the structural regression model, the indirect effects of procedural justice on violent misconduct via risky lifestyles were examined. Figure 1 presents the structural regression model in the current study. Table 3 shows the standardized coefficients from the structural regression model. All item loadings on three latent variables were statistically significant (*p* < 0.001), and the overall model fitted the data: *χ*^2^ = 138.445, df = 72, *p* < 0.001; GFI = 0.979; CFI = 0.984; and RMSEA = 0.029, 95% CI = [0.015, 0.043].

As hypothesized, procedural justice had a negative and significant direct effect on risky lifestyles (β = −0.178, *p* < 0.001). Additionally, risky lifestyles had a positive and significant effect on violent misconduct (β = 0.573, *p* < 0.001). The effect of procedural justice on violent misconduct was also found to be significant. The findings suggested that procedural justice affected violent misconduct not just directly but also indirectly via opportunities created from risky lifestyles in prison settings. The results of the indirect effect indicated that higher levels of procedural justice led to lower levels of violent misconduct through its impact on risky lifestyles, even after controlling for all other covariates (β = −0.147, *p* < 0.001).

Other covariates, age (β = −0.128, *p* < 0.001), length of time served (β = 0.181, *p* < 0.001), and psychological treatment (β = 0.100, *p* < 0.05) were significant predictors of risky lifestyles. Yet, only two covariates, education (β = −0.091, *p* < 0.05) and work in prison (β = −0.090, *p* < 0.05), were significant predictors of violent misconduct. Overall, the full model explained 38% of the variance in violent misconduct.

### Supplementary Analyses

**Figure 2:**
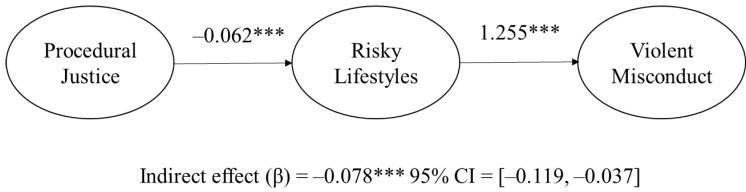
The indirect effect of procedural justice on violent misconduct via risky lifestyles. Standard coefficients are presented. CI = confidence interval. *** *p* < 0.001.

To test the robustness of the predictors, a series of supplementary analyses were conducted. First, we re-estimated the model after removing non-significant paths. All predictors retained their statistical significance. Second, the indirect effect was examined based on the bias-corrected bootstrapped CIs [91]. Given that several covariates included in the model were skewed, the use of bootstrapping provides more accurate estimates to evaluate the indirect effects of procedural justice. Thus, 95% CIs were estimated with the 5000 bootstrap samples of indirect effects. The results of the bootstrapping are presented in Figure 2. The findings supported the research hypothesis that the relationship between procedural justice and violent misconduct is mediated by risky lifestyles, as shown by the exclusion of 0 from the 95% CIs for the indirect effect (β = −0.078, *p* < 0.001, *SE* = 0.021, 95% CI = [−0.119, −0.037]).

## 5. Discussion

A recent focus of procedural justice research has centered on the mechanism through which procedural justice influences individuals’ compliance with the law [13,14,92,93]. More specifically, this line of work has explored the mediating role of social identity in linking procedural justice to obedience to the law [22]. In this article, we explored another potential mechanism that may help to account for the differences linking procedural justice to violent misconduct. We suggest that one promising perspective resides in the opportunity perspectives: low procedural justice may lead people to self-select into risky lifestyles. As Gottfredson [94] noted, “the processes that reduce the restraints to offend are similar to the processes in lifestyle terms that affect the probability that persons will be places at times and around people where the risk of victimization is high” (p. 726). Considering that the absence of procedural justice can lower the level of self-regulating beliefs [1,95,96], the logic of procedural justice may suggest that procedurally unfair treatment at the hands of legal agents can promote risky lifestyles. In the current study, we examined the extent to which a higher level of procedural justice influences inmates’ risky lifestyles and violent misconduct. Our research, using data from a sample of serious South Korean offenders, yielded two key findings.

First, our findings indicated that risky lifestyles were associated with procedural justice in addition to the length of time served, the number of times in prison, and low self-control. These findings suggest that the role of procedural justice lies not only in developing a social identity that facilitates self-regulation but also in curbing risky lifestyles that may expose individuals to the settings that can entice, enable, or encourage more serious offending [25]. Interestingly, the length of time served was predictive of risky lifestyles. Given that offenders can develop “cognitive maps” or images of their surroundings that can guide potential offenders to engage in risky activities [97], the accumulated experience of prison can broaden the inmates’ “awareness space” that offer opportunities for behaviors that violate institutional regulation [98].

Second, in analyses designed to examine the mediating role of risky activities in the association between procedural justice and violent misconduct, the results showed that risky activities act as a mediator linking procedural justice and violent offending. Previous studies have shown that social identity serves as an important social-psychological bridge in the association between procedural justice and obedience with the law, but it is only a partial mediator [13,14]—implying that other variables may help explain how procedural justice indirectly influences compliance with the law. Our findings suggest that opportunity perspectives can offer a theoretical lens through which to view perceived procedural justice and subsequent violent misconduct.

### 5.1. Theoretical and Policy Implications

The application of the opportunity framework in understanding the role of procedural justice is not necessarily incompatible with the insights from the procedural justice theory, which emphasizes the internalization of group values and norms when individuals are being treated fairly by criminal justice agents [2,22]. The reason why inmates who perceive fair treatment did not participate in risky lifestyles might be that fairness reinforced the social bonds between inmates and the prison institution, which led them to abide by institutional regulations. Consequently, inmates with a higher level of perceived procedural justice tended to be less involved in risky lifestyles that are at odds with institutional rules. Thus, our findings complement the findings from the line of work that focuses on the link between procedural justice and compliance with the law [13,93].

Although these results reflect only one study, if future research replicates these findings, it will be critical for crime control policies to incorporate efforts not only to enhance the quality of treatment by criminal justice agents, including correctional officers, but also to reduce criminal opportunities associated with risky lifestyles [99]. A wealth of research has shown that procedural justice can deter crime among not only the general population but also offender populations [8,9,10]. The current results from a sample of incarcerated offenders suggest that attempts to provide a high quality of treatment at the hand of correctional officers may serve to help inmates’ adjustment to prison [63,64]. Moreover, the current results highlight the importance of continued attempts to keep inmates away from situations that would lure them into more serious crimes [25]. Effective supervision should involve identifying how inmates participate in risky lifestyles and blocking inmates from crime-conducive settings. It would also be beneficial to consider situational crime prevention that can make risky lifestyles more difficult and less rewarding [100]. For example, improving the quality of security technology can reduce the number of blind corners or recess, in which inmates can engage in risky activities.

### 5.2. Study Limitations

Our study was limited in a few respects. First, the current study used cross-sectional/retrospective data to explore the proposed relationships between procedural justice, risky lifestyles, and violent misconduct. The use of current measures of procedural justice and risky lifestyles to predict past-year offending create temporal order concerns. Subsequent research should employ longitudinal data to measure perceptions at one-time period and inmate misconduct at a later time period to strengthen the current findings.

Another limitation involves the measurement of procedural justice. There are various ways of measuring the concept of procedural justice [20,101,102]. Although our measure of procedural justice can be situated within the mainstream literature and operated as predicted [55], data collection efforts should be continued to empirically examine the relationship between procedural justice and risky lifestyles with more robust measures of procedural justice. Relatedly, the current study could not use measures of procedural justice regarding other agents of the criminal justice system but correctional officers. Some researchers noted that there can be spill-over effects of perceptions of procedural justice between different elements of the criminal justice system [103]. We call for future research to examine the issues regarding spill-over effects in the correctional setting.

Furthermore, we constructed the measures based on self-report data collected from a single source (i.e., the inmates), and this can raise an issue involving common method variance [104]. However, it should be noted that official institutional records in prisons also have their limits because correctional officers do not report all rule-breaking behaviors by inmates that they observed [105]. Importantly, we did not include key individual characteristics of inmates that could have influenced the patterns of behaviors and responses reported in the study. For example, low self-control is an essential individual trait known to be associated with crime and risky lifestyles [106,107]. Although the focus of the current study was not the mechanism through which low self-control is related to violent misconduct, we suggest that future research on the topic of procedural justice in a correctional setting can consider key individual traits to clarify the causal chains between these concepts.

Finally, although we explored the mediating role of risky lifestyles, we could not include other mechanisms that have been shown to link procedural justice and compliance with the law (e.g., social identity) [14,60]. Empirical investigations should be pursued to clarify the underlying theoretical processes through which procedural justice carries its effect on compliance with the law.

### 5.3. Conclusions

Notwithstanding the limitations, our study, using a sample of South Korean inmates who were imprisoned for serious crimes, highlights the role of risky lifestyles in the association between procedural justice and violent misconduct. Our research also uncovered that accumulated experiences in prison can promote risky lifestyles. Studies like ours and future ones are important for theoreticians, policymakers, and the general public to have a better understanding of the mechanisms through which improvements in procedural justice can benefit the criminal justice system to help develop theoretical frameworks and prevention/intervention efforts that are based on scientific findings.

## Figures and Tables

**Table 1:** Study sample descriptive statistics (N = 986).

Variable	M	%	SD	Minimum	Maximum
***Violent misconduct***					
Fighting with fellow inmates	0.36	—	0.65	0	4
Assaulting fellow inmates	0.17	—	0.52	0	4
Assaulting correctional officers	0.03	—	0.21	0	4
***Procedural justice***					
Trustworthiness	2.54	—	0.84	1	4
Respect	2.36	—	0.84	1	4
***Risky Lifestyle***					
Possessed prohibited items	—	14.99	—	0	1
Broke away from the designated area	—	8.02	—	0	1
Gambled	—	14.59	—	0	1
Transaction	—	3.90	—	0	1
***Other Covariates***					
Age	39.25	—	10.28	19	74
Education	2.85	—	0.91	1	5
Marital status (single, bereaved, divorced = 1)		70.05	—	0	1
Length of time served (logged)	3.25	—	0.96	0.51	6.18
Academic education	—	26.55	—	0	1
Vocational training	—	25.77	—	0	1
Psychological treatment	—	12.89	—	0	1
Work in prison	—	62.65	—	0	1

Abbreviation: M = Mean, SD = standard deviation.

**Table 2:** The factor loadings of the measurement model.

Latent Variables	Observed Indicators	Factor Loading
Violent misconduct	Fighting with fellow inmates	0.766 ***
	Assaulting fellow inmates	0.729 ***
	Assaulting correctional officers	0.483 **
Procedural justice	Trustworthiness	0.750 ***
	Respect	0.953 ***
Risky Lifestyle	Possessed prohibited items	0.598 ***
	Broke away from the designated area	0.694 ***
	Gambled	0.570 ***
	Transaction	0.527 ***
Chi-square test of model fit (*χ*^2^) df = 24		28.832
GFI		0.997
CFI		0.987
RMSEA		0.015

Note. ** *p* < 0.01; *** *p* < 0.001. Abbreviations: GFI = goodness-of-fit index; CFI = comparative fit index; TLI = Tucker–Lewis index; and RMSEA = root mean square error of approximation.

**Table 3:** Summary of OLS regression coefficients.

Variable	Risky Lifestyles		Violent Misconduct	
	β	(*SE*)	β	(*SE*)
Procedural justice	−0.178 ***	(0.016)	−0.090 *	0.032
Risky Activities	—	—	0.573 ***	0.133
Age	−0.128 ***	(0.001)	−0.039	0.002
Education	0.001	(0.011)	−0.091 *	0.021
Marital status	−0.034	(0.021)	−0.029	0.041
Length of time served (logged)	0.181 ***	(0.011)	0.029	0.022
Academic education	0.020	(0.024)	−0.013	0.047
Vocational training	−0.039	(0.024)	−0.053	0.047
Psychological treatment	0.100 *	(0.028)	0.046	0.056
Work in prison	0.073	(0.020)	−0.090 *	0.038
*R* ^2^	0.114		0.381	

Note: OLS = ordinary least squares; *SE* = standard error. * *p* < 0.05, *** *p* < 0.001 (two-tailed tests).
